# Spatially resolved electrochemiluminescence through a chemical lens†

**DOI:** 10.1039/d0sc04210b

**Published:** 2020-09-14

**Authors:** Andrea Fiorani, Dongni Han, Dechen Jiang, Danjun Fang, Francesco Paolucci, Neso Sojic, Giovanni Valenti

**Affiliations:** Department of Chemistry “G. Ciamician”, University of Bologna Via Selmi 2 40126 Bologna Italy g.valenti@unibo.it; Univ. Bordeaux, Bordeaux INP, ISM, UMR CNRS 5255 33607 Pessac France neso.sojic@enscbp.fr; School of Pharmacy, Key Laboratory of Targeted Intervention of Cardiovascular Disease, Collaborative Innovation Center for Cardiovascular Disease Translational Medicine, Nanjing Medical University Nanjing Jiangsu 211126 China; State Key Laboratory of Analytical Chemistry for Life Science, School of Chemistry and Chemical Engineering, Nanjing University Nanjing Jiangsu 210093 China; Department of Chemistry, South Ural State University Chelyabinsk 454080 Russian Federation

## Abstract

Electrochemiluminescence (ECL) microscopy is an emerging technique with a wide range of imaging applications and unique properties in terms of high spatial resolution, surface confinement and favourable signal-to-noise ratio. Despite its successful analytical applications, tuning the depth of field (*i.e.*, thickness of the ECL-emitting layer) is a crucial issue. Indeed, the control of the thickness of this ECL region, which can be considered as an “evanescent” reaction layer, limits the development of cell microscopy as well as bioassays. Here we report an original strategy based on chemical lens effects to tune the ECL-emitting layer in the model [Ru(bpy)_3_]^2+^/tri-*n*-propylamine (TPrA) system. It consists of microbeads decorated with [Ru(bpy)_3_]^2+^ labels, classically used in bioassays, and TPrA as the sacrificial coreactant. In particular we exploit the buffer capacity of the solution to modify the rate of the reactions involved in the ECL generation. For the first time, a precise control of the ECL light distribution is demonstrated by mapping the luminescence reactivity at the level of single micrometric bead. The resulting ECL image is the luminescent signature of the concentration profiles of diffusing TPrA radicals, which define the ECL layer. Therefore, our findings provide insights into the ECL mechanism and open new avenues for ECL microscopy and bioassays. Indeed, the reported approach based on a chemical lens controls the spatial extension of the “evanescent” ECL-emitting layer and is conceptually similar to evanescent wave microscopy. Thus, it should allow the exploration and imaging of different heights in substrates or in cells.

## Abstract 2

A versatile mechanism based on a chemical lens to control the electrochemiluminescence (ECL) spatial distribution is presented. Changing the buffer capacity modifies the rate of ECL reactions, and therefore the thickness of the ECL-active layer.

## Graphical abstract

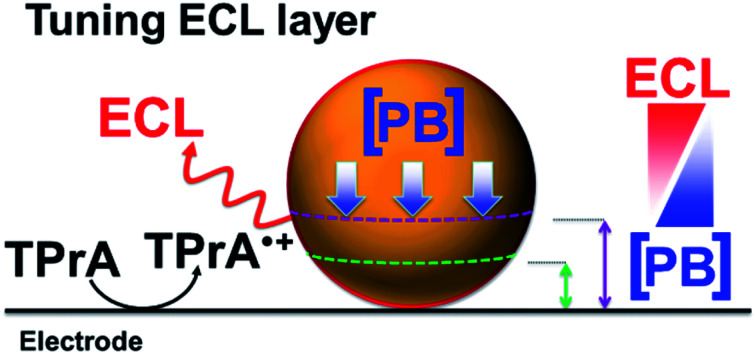

## Introduction

**Scheme 1:**
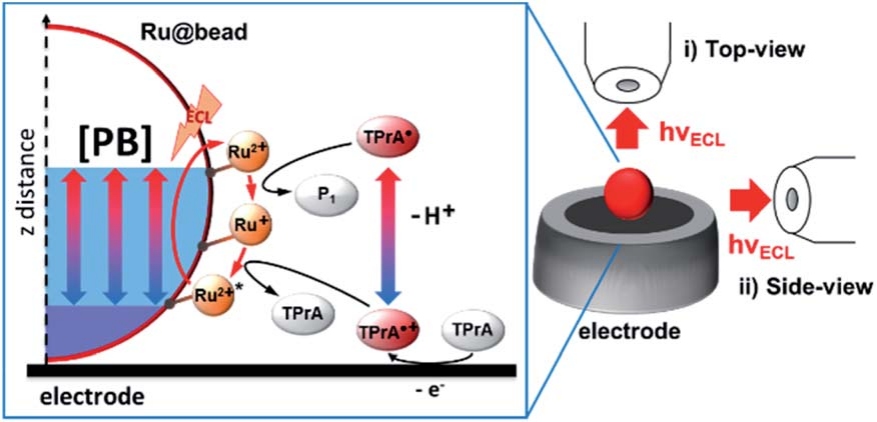
Schematic description of the chemical lens strategy applied to the ECL mechanism of a microbead (Ru@bead) functionalized with the ECL luminophore ([Ru(bpy)_3_]^2+^), here denoted as Ru^2+^. The tri-*n*-propylamine (TPrA) coreactant is oxidized at the electrode generating the cation radical (TPrA˙^+^) that deprotonates to give the neutral radical (TPrA˙). The neutral radical and then the cation radical react sequentially with [Ru(bpy)_3_]^2+^ immobilized on the magnetic beads and generate its excited state. [PB] indicates a variation of the phosphate buffer concentration that allows controlling the thickness of the ECL-emitting layer. On the right, both optical configurations used to image the functionalized beads are presented: (i) top view and (ii) side view. P1 is the product of homogeneous TPrA˙ oxidation with [Ru(bpy)_3_]^2+^.

Electrochemiluminescence (ECL) is the light emission induced by an initial electrochemical reaction at the electrode surface. It has been widely investigated and has found successful applications in various fields ranging from fundamental studies on highly exergonic electron-transfer reactions, biosensing, and environmental chemistry to microscopy.^1–5^ Nowadays, ECL is a leading transduction technique with important applications in the early diagnosis of many diseases thanks to its unique signal-to-noise ratio.^6–11^ Besides the regular analytical applications, the combination of ECL with microscopy (ECLM) is an emerging technique that provides high lateral resolution, typical of microscopy, with surface-confined processes since the emission is restricted within the ECL reaction layer (*i.e.*, μm range near the electrode surface).^12–14^ Recently, we reported the first example of ECLM for the visualization of single cells and detection of cancer biomarkers on the cellular membrane.^15–17^ Different approaches have also been proposed to image cells by ECLM.^12,13,18–22^ Furthermore, the surface-confined emission allows ECLM to quantify different analytes simultaneously^23–26^ as well as permitting high resolution visualization of nanomaterials such as nanoparticles and nanorods.^27–31^ In ECL, the emission layer is typically confined in the near proximity of the electrode surface^32^ and it is limited by the lifetime of the coreactant radicals (*vide infra*) and cannot be easily controlled.^33^ Different strategies have been proposed to extend the ECL emitting layer including the application of the so-called Faraday cage^34^ and the choice of different coreactants.^35^ However, most of these approaches are limited and a versatile method is still an open challenge. This issue restricts the development of ECLM as well as the sensitivity of bioassays because it does not allow extension of the ECL layer and imaging of objects or subcellular structures located a few microns away from the electrode surface. Here, we report a novel concept based on a combination of ECLM and a chemical lens for the control of the spatial extension of the ECL-emitting layer (Scheme 1).

In a pioneering study, Heinze and co-workers introduced the concept of chemical lens to control the concentration profiles of electrogenerated species.^36,37^ A chemical lens was proposed to increase the lateral resolution of scanning electrochemical microscopy during electrodeposition in order to downsize the features of a patterned surface.^38^ This approach involved the use of an additional species that does not take part in the main reaction but rather scavenges one of its by-products. By changing the concentration of the scavenger, the reaction layer of electrogenerated species can be shrunk to achieve higher spatial resolution.

In the ECL mechanism, the by-product that does not take part directly in the luminescent reaction is the proton (eqn (1)–(5)). This species is released during the deprotonation step of the oxidized form of TPrA (*i.e.*, TPrA˙^+^), which generates the neutral TPrA˙ radical (eqn (2)). The chemical confinement of H^+^ can in principle be controlled by the buffer capacity of the supporting electrolyte.^39,40^ The overall effect will result in the modulation of the concentration profiles of both TPrA radicals, *i.e.*, change of the ECL-active region. Indeed, the spatial distribution of ECL is the luminescent signature of the concentration profiles of both diffusing TPrA radicals, which react with the immobilized [Ru(bpy)_3_]^2+^ and thus control the extension of the ECL-emitting layer.

Herein, the distribution of the ECL-emitting layer has been investigated by ECLM at the level of single microbeads with different dimensions (8, 12 and 14 μm). The model ECL [Ru(bpy)_3_]^2+^ label was conjugated to the beads (named Ru@bead) resembling bead-based immunoassays^17,41–43^ (see the ESI for the details†). ECL emission was generated in a configuration named surface generation–bead emission.^14^ This approach could be used as a model system for the ECLM of real samples such as cells, and also to mimic the analytical approach of commercial ECL-based immunoassay systems.^41^ The radicals are generated by coreactant oxidation at the electrode surface and freely diffuse in solution, while the ECL emission comes from the labelled microbeads. In this context, the microbead acts as a probe to investigate the reactivity of the coreactant radicals, which allows mapping the active ECL-emitting layer as a function of the proton availability.^44^

## Results and discussion

In the present work, we investigated the model ECL system tris(2,2′-bipyridine)ruthenium(ii) ([Ru(bpy)_3_]^2+^) as the light-emitting species and TPrA as the sacrificial coreactant, which follows an “oxidative-reduction” path. It provides high ECL efficiency in heterogeneous as well as homogeneous formats and it is the most widely exploited ECL system to detect various biomolecules (*e.g.*, proteins, peptides, ligands and oligonucleotides) and DNA, for immunological assays and ECL microscopy.^11–22^ The heterogeneous ECL mechanism of the [Ru(bpy)_3_]^2+^/TPrA system is an active area of investigation.^14,32,33,43,44^ In such a configuration, the ECL luminophore is not free to diffuse and cannot be directly oxidised at the electrode.^45,46^ In the current system under investigation, the [Ru(bpy)_3_]^2+^ label is covalently bound to the bead as in bead-based ECL immunoassays. Since the bead is made of an insulating material, only an infinitesimal fraction of the [Ru(bpy)_3_]^2+^ complex, within electron tunnelling distance from the electrode surface, could be directly oxidised.^47^ Considering the respective micrometric and nanometric dimensions of the bead and of the tunnelling distance, ECL resulting from the direct oxidation of [Ru(bpy)_3_]^2+^ can be neglected.^14,33,45,48^ The ECL is induced exclusively by the direct oxidation of the freely diffusing TPrA (eqn (1)) that, upon oxidation, partially undergoes a deprotonation reaction (eqn (2)). Thus, it forms a high energetic radical species^49^ able to reduce the ECL luminophore [Ru(bpy)_3_]^2+^ to [Ru(bpy)_3_]^+^ (eqn (3)). On the other hand, the pristine oxidized coreactant is continuously produced at the electrode surface, and it can react with [Ru(bpy)_3_]^+^ to generate the excited state [Ru(bpy)_3_]^2+^* (eqn (4)).^32,44^ Finally, [Ru(bpy)_3_]^2+^* relaxes to the ground state generating the ECL signal (eqn (5)). The general equation scheme is as follows:

1 TPrA − e^−^ → TPrA˙^+^

2 TPrA˙^+^ ⇆ TPrA˙ + H^+^

3 TPrA˙ + [Ru(bpy)_3_]^2+^ → P1 + [Ru(bpy)_3_]^+^

4 TPrA˙^+^ + [Ru(bpy)_3_]^+^ → TPrA + [Ru(bpy)_3_]^2+^*

**Fig. 1:**
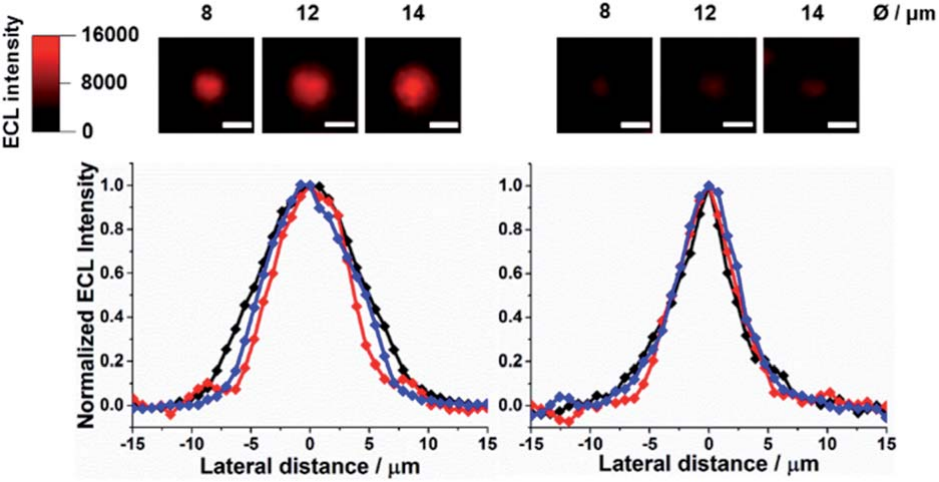
Top-view ECL images of Ru@beads in 0.01 M PB (left) and 1 M PB (right), and their respective normalized ECL profiles extracted from the bead imaging: 8 μm (red), 12 μm (blue) and 14 μm (black). Scale bar: 10 μm.

5 [Ru(bpy)_3_]^2+^* → [Ru(bpy)_3_]^2+^ + *hν*

where P1 is the product of the homogeneous TPrA˙ oxidation. Eqn (2) has been recently proposed by Amatore and co-workers as a pseudo first order reaction at equilibrium.^44^ Due to the limited lifetime of TPrA˙^+^, this mechanism is active at very short distances from the electrode surface, and is the only pathway able to generate ECL when the ruthenium label is immobilized on magnetic beads, on cells or in sandwich immunoassays.^46^ Many research groups, including ours, mapped the ECL reactivity and the emission spatial distribution in such a context.^14,15,33,43^ The maximum of ECL emission occurs in the micrometric region where concentrations of TPrA˙ and TPrA˙^+^ radicals are locally the highest.^43,44^ We also demonstrated, by analysing the side-view ECL image of the beads that only luminophores located within the 3 μm region close to the electrode contribute to the signal.^33^ The diffusion profiles of TPrA˙ and TPrA˙^+^ radicals are determined by the lifetime of TPrA˙^+^ in turn imposing the space region where the ECL emission is obtained. Noteworthily, from the general equation scheme (eqn (1)–(5)), we recognize the essential role of the proton-accepting base in defining the concentration gradient ratio of [TPrA˙]/[TPrA˙^+^], as described by the pseudo first order deprotonation reaction (eqn (2)).^14,32,44,50^ Therefore, we analysed the ECL emission from Ru@bead with a top-view configuration (Scheme 1) with different phosphate buffer (PB) concentrations (Fig. 1 and S1†). We demonstrated that the ECL signal from Ru@bead increased with lower PB concentrations. The measurement of the ECL emission from microbeads of different dimensions and at different concentrations of PB confirms that eqn (2) is an equilibrium process (eqn (2′)) whose position is critically determined by the availability of proton-accepting phosphate ions.

2′ TPrA˙^+^ + PO_4_^3−^ ⇆ TPrA˙ + HPO_4_^2−^

Fig. 1 shows that the profile of ECL emission in a top-view configuration from 8 μm, 12 μm, and/or 14 μm microbeads shrinks significantly upon increasing the PB concentration (0.01, 0.1 and 1 M Fig. S1†), in line with the proton scavenging ability of phosphate ions. This effect would make the radical cation less available for promoting ECL through eqn (4). The full width at half-maximum (FWHM) of the ECL intensity profiles measured on the labelled beads decreases by ≈43% and ≈27%, for 12–14 μm and 8 μm microbeads, respectively, reaching the same value of about 6–5.5 μm for 1 M PB (Fig. S2†).

**Fig. 2:**
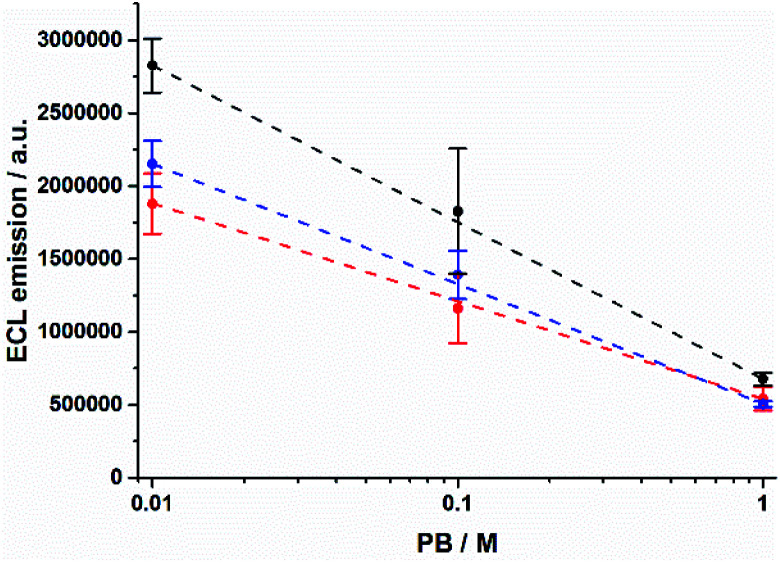
Integrated ECL emission as a function of PB concentration for 8 μm (red), 12 μm (blue), and 14 μm (black) Ru@beads. Error bars show the standard deviation (*n* = 4). Lines are only a guide to the eye.

The shrinking of the ECL profiles is also reflected in the total ECL emission from the microbeads (Fig. S3†). The ECL integral decreases as PB concentration increases (Fig. 2). We observed a decrease of ≈76% and ≈71% for 12–14 μm and 8 μm beads, respectively. In contrast, the anodic current due to the TPrA oxidation shows an opposite behaviour demonstrating that different phosphate buffer concentrations do not hinder the TPrA oxidation (Fig. S4†).

**Fig. 3:**
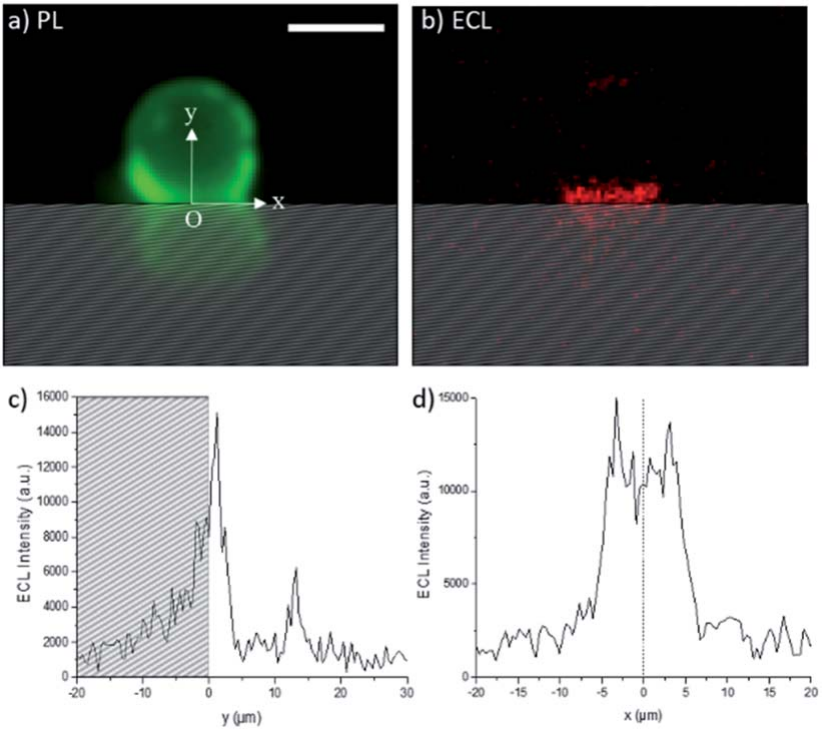
Side-view images of a 12 μm polystyrene bead labelled with the [Ru(bpy)_3_]^2+^ complex recorded in 0.01 M PB solution using (a) PL (green) and (b) ECL (red) modes. Experimental ECL intensity profiles along the (c) vertical *y*-axis and (d) horizontal *x*-axis. The hatched zone represents the luminescence reflection on the electrode surface. The origin O of the two axes corresponds to the electrode surface (*y* = 0) and to the middle of the bead (*x* = 0). The *y*-axis is the symmetry axis passing through the middle of the bead. Applied potential: 1.4 V *vs.* Ag/AgCl. Scale bar: 10 μm.

The ECL images recorded in the top-view configuration do not provide very precise information on the extension of the ECL-emitting layer since the detected ECL light has to pass through the bead, which modifies the optical paths. Indeed, the bead may act as an optical lens.^51^ In addition, as pointed out by Amatore *et al.*, the effect of intrinsic distortions, ascribed to optical imaging of the confocal volume of ECL generation, results in a widening of the recorded image,^52^ and the broadening of the ECL image is affected by the focal plane of sampling. To further investigate the effects of the PB concentration on the thickness of the ECL-emitting layer, we changed the angle of observation from the top-view to a side-view configuration (Scheme 1). This optical configuration supplements the top-view mapping with a 2D imaging. Fig. 3 shows the photoluminescence (PL) and ECL images of the same single bead modified with the [Ru(bpy)_3_]^2+^ labels. The PL imaging shows the real bead (upper part of the image) and its mirror image (lower part); this latter is formed by the PL reflection on the electrode surface. For the sake of clarity, the image obtained by reflection is materialized with the hatched zone. The position of the bead and also its interface with the electrode are precisely defined using the PL image. The *x* and *y* axes are parallel and normal to the electrode surface, respectively. We set the origins of both *x* and *y* axes at the electrode surface (*y* = 0) where the bead is in contact with the electrode (*x* = 0). In other words, at *x* = 0, the *y*-axis is the symmetry axis passing through the middle of the bead. As reported previously,^33^ two micrometric ECL-emitting zones are visible in Fig. 3b: (i) the one close to the electrode reflects the ECL reactivity while (ii) the one located at the top of the bead corresponds to the focusing effects of the bead which acts as an optical lens. The real chemical information is contained only in the ECL region located close to the electrode surface. From Fig. 3b, we extracted the ECL intensity profiles along both *y* and *x* axes (Fig. 3c and d, respectively). As described in a previous report,^33^ the ECL-emitting region extends only over 3–4 μm along the vertical axis, which is normal to the electrode surface (Fig. 3c). The FWHM of this ECL intensity profile is 3.1 μm. In Fig. 3c, the small ECL peak located at *y* ≈ 12 μm corresponds to the already mentioned optical lens effects of the bead, which is based on a completely different physical phenomenon from the chemical lens effects that we aim to study in this work. The ECL intensity profile along the *x*-axis is displayed in Fig. 3d. The shape is symmetric around the fixed point O.

**Fig. 4:**
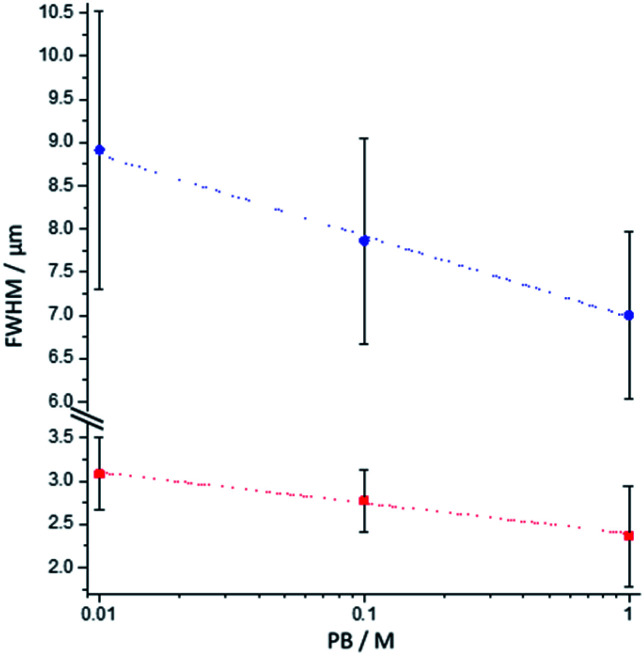
Effect of the PB concentration on the FWHM values measured along the *x*-axis (blue dots) and the *y*-axis (red squares) of the bead as defined in Fig. 3a. The values were extracted from the side-view images of single 12 μm [Ru(bpy)_3_]^2+^-decorated polystyrene beads. Error bars show the standard deviation (*n* = 9). Lines are only a guide to the eye.

To analyse further the chemical lens effects of the PB concentration on the volumic extension of the ECL-emitting region, we used the side-view configuration to record the ECL images of the [Ru(bpy)_3_]^2+^-decorated beads and extracted the FWHM values from the ECL intensity profiles along both axes (Fig. 4). Increasing the PB concentration from 0.01 M to 1 M causes the decrease of the thickness of the ECL-emitting layer. This is evidenced by the average values of the FWHM along the *y*-axis, which decrease from 3.1 μm to 2.4 μm (Fig. 4, red squares). The same trend is also noticed along the *x*-axis with a progressive decrease of FWHM from 8.9 μm to 7 μm (Fig. 4, blue dots). This evolution is consistent with the behaviour observed in the top-view configuration and previously reported simulations.^44^

The whole body of experimental evidences is in agreement with a diffusion profile of TPrA˙^+^ constricted nearer to the electrode surface with increasing PB concentration. This is a consequence of readily available phosphate ions for buffering the hydrogen ions released from TPrA˙^+^ deprotonation, thus resulting in a shortened TPrA˙^+^ diffusion length. This will reflect in a narrower emission profile, associated with a lower ECL emission, since a smaller area of the microbead is reached by the diffusing TPrA˙^+^ which triggers the reactions in Scheme 1 (eqn (1)–(5)).

## Conclusions

In summary, we have presented here a simple method to modify the ECL layer and also to provide insights into the ECL mechanism. Our strategy, which is compatible with bioanalytical detection and experiments on cells, has a direct impact on the ECL spatial distribution. Indeed, by changing the buffer capacity, we were able to modify the thickness of the ECL-emitting layer. Overall, our report paves the way for imaging different heights in substrates or in single cells in an approach conceptually similar to total internal reflection fluorescence microscopy (TIRFM) where we can control the “evanescent” ECL-emitting layer with a chemical lens.

## Conflicts of interest

There are no conflicts to declare.

## Supplementary Material

SC-011-D0SC04210B-s001
