# Supplementary material for: Spatially resolved electrochemiluminescence through a chemical lens
Source: Chem Sci. 2020 Sep 14;11(38):10496–500. doi: 10.1039/d0sc04210b (PMC8162283; doi:10.1039/d0sc04210b)
Supplement: SC-011-D0SC04210B-s001 [file SC-011-D0SC04210B-s001.pdf]

Electronic Supplementary Material (ESI) for Chemical Science.
This journal is © The Royal Society of Chemistry 2020

Supporting Information for

Spatially Resolved Electrochemiluminescence Through Chemical Lens

Andrea Fiorani,[‡a] Dongni Han,[bc] Dechen Jiang,[d] Danjun Fang,[c] Francesco Paolucci,[a] Neso Sojic[*b,e] and Giovanni Valenti[*a]

a. University of Bologna, Department of Chemistry "G. Ciamician", via Selmi 2, 40126, Bologna, Italy.

b. Univ. Bordeaux, Bordeaux INP, ISM, UMR CNRS 5255, 33607 Pessac, France. E-mail:

c. School of Pharmacy and Key Laboratory of Targeted Intervention of Cardiovascular Disease, Collaborative Innovation Center for Cardiovascular Disease Translational Medicine, Nanjing Medical University, Nanjing, Jiangsu 211126, China.

d. State Key Laboratory of Analytical Chemistry for Life Science and School of Chemistry and Chemical Engineering. Nanjing University. Nanjing, Jiangsu, 210093, China.

e. Department of Chemistry, South Ural State University, Chelyabinsk 454080, Russian Federation.

[‡]Current address: Department of Chemistry, Keio University, 3-14-1 Hiyoshi, 223-8522, Yokohama, Japan

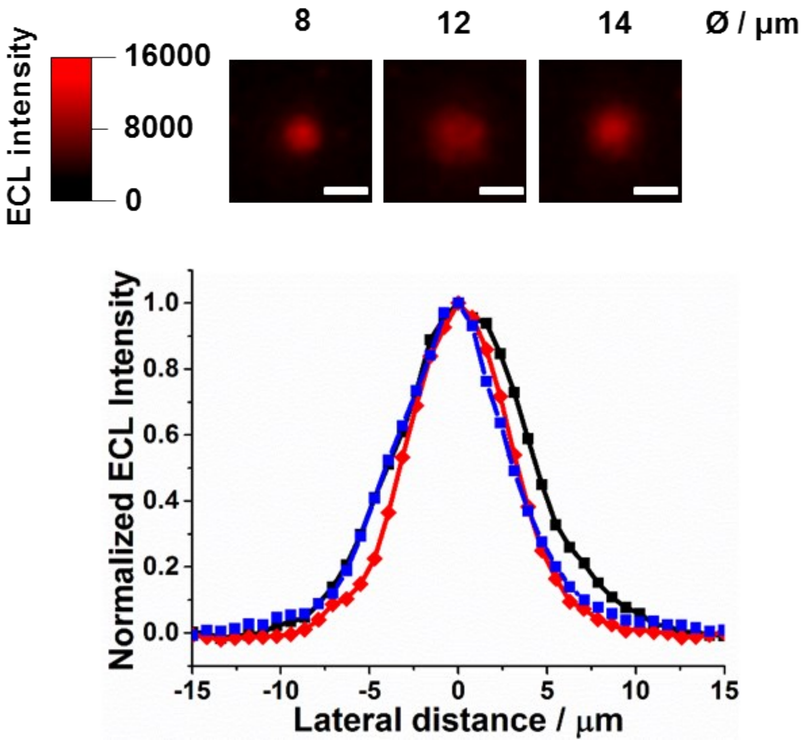

Fig. S1 Top: ECL images of 8 µm, 12 µm and 14 µm microbead labeled with the $[Ru(bpy)_3]^{2+}$ complex recorded in 0.1 M PB by top-view configuration. Bottom: normalized ECL profile of beads for PB 0.1 M, red trace are 8 µm beads, blue trace are 12µm, and black trace are 14 µm beads. Applied potential: 1.4 V vs. Ag/AgCl. Scale bar: 10 µm.

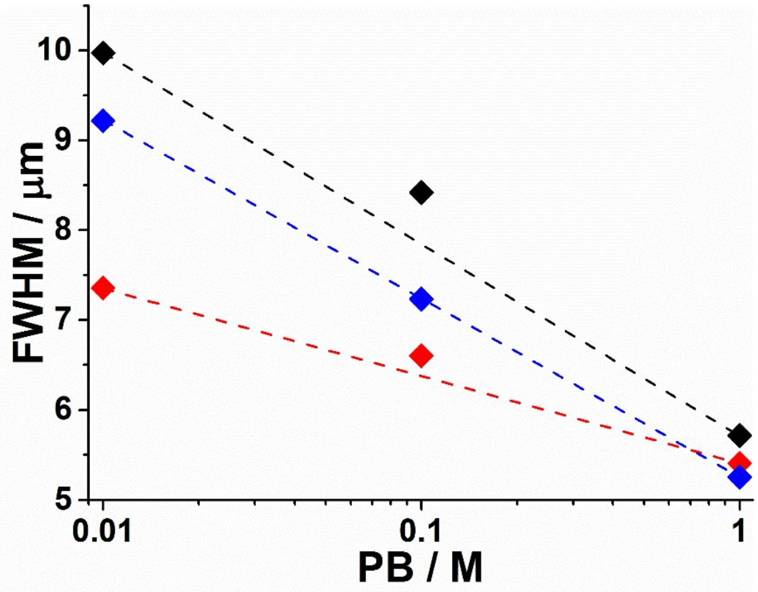

Fig. S2 Full width at half maximum of microbead profiles as function of PB concentration (from Figure 1 and S2) for 8 µm (red), 12 µm (blue), and 14 µm (black).

MATERIALS AND METHODS

Chemicals. All chemicals were obtained from Sigma-Aldrich, unless otherwise stated, and were used as received. Beads were from Spherothech (USA) 8 and 14 µm, and Kisker Biotech GmbH & Co. (Germany) 12 µm.

Preparation of $[Ru(bpy)_3]^{2+}$ labelled beads (8, 12 and 14 µm) used in top-view ECL imaging. This procedure has been described in detail elsewhere.[1]

Briefly, a solution of bis(2,2'-bipyridine)-[4-(4'-methyl-2,2'-bipyridin-4-yl)butanoic acid] ruthenium bis(hexafluorophosphate) ($Ru(bpy)_3^{2+}$–COOH, Cyanagen, Italy) in DMF (70 µL, 0.47mg) was added to 1.5 equivalents of N,N'-dicyclohexylcarbodiimide (DDC) and mixed for 4 h at room temperature. A streptavidin solution (630 µL, 0.665 mg) in 0.1 M borate buffer (pH 9.4) was added to the activated $Ru(bpy)_3^{2+}$–COOH and incubated overnight. The labelled streptavidin was dialyzed against 5 L of PBS 1X. COOH-functionalized beads (200 µL) were washed three times in 0.1 M borate buffer (pH 9.6) and two times in 0.1 M in 2-(N-morpholino)ethanesulfonic acid buffer (MES pH 5.5), and finally re-suspended in 250 µL of MES buffer. The beads solution was added with N-(3-dimethylaminopropyl)-N'-ethyl-carbodiimide hydrochloride (EDC) and hydroxy-2,5-dioxopyrrolidine-3-sulfonicacid sodium salt (sulfo-NHS) to a final concentration of 50 mM and 2 mM, respectively, and mixed for 1 h at room temperature. After one washing with MES, biotin cadaverine (500 µL, 9 mM) in 0.1 M borate buffer (pH 8.6) were added and incubated overnight at 48°C. The beads solution was finally washed three times in PBS. Buffer was removed from 50 µL of beads and 50 µL of labelled $Ru(bpy)_3^{2+}$–streptavidin were added and mixed for 2 h at room temperature, followed by three washing steps and re-suspension with PBS.

Preparation of $[Ru(bpy)_3]^{2+}$ labelled beads (12 µm) used in side-view ECL imaging. This procedure has been described in detail elsewhere.[2]

10 µL of NH_2-functionlized beads were washed with PBS (1X, pH=7.4) and re-suspended in 1 mL of PBS. 1 mg of (bis(2,2'-bipyridine)-4'-methyl-4-carboxybipyridine-ruthenium N-succinimidyl ester-bis(hexafluorophosphate) ($Ru(bpy)_3^{2+}$-NHS ester) was dissolved in 100 µL of dimethyl sulfoxide and added to the beads suspension. This mixture was incubated at 4°C for 3 hours with continuous stirring. After the incubation the beads were washed with PBS and re-suspended in 1 mL PBS.

Top-view ECL imaging: The ECL imaging was performed in a PTFE homemade electrochemical cell in a three electrode configuration, glassy carbon working electrode, Ag/AgCl (KCl, 3M) reference electrode and Pt wire counter electrode. ECL images were acquired by an epifluorescence microscope from Nikon equipped with an Hamamatsu Electron Multiplying Charge Coupled Device (EM-CCD) with a resolution of 512 × 512 pixels and long distance objectives from Nikon (20 × /0.40 DL13 mm). The microscope was enclosed in a homemade dark box to avoid interferences from external light, and electrochemical cell positioning was assisted by a motorized microscope stage Corvus Märzhauser. The system included a potentiostat Metrohm PGSTAT 30 for electrochemical measurements.

ECL was triggered by applying 1.4 V for 8 s, and the emission collected by EM-CCD. The pH of all PB solutions has been adjusted to 7.4 by addition of concentrated phosphoric acid and the TPrA concentration was 180 mM.

The ECL emission from top-view ECL images was integrated by ImageJ software. Briefly, a .TIFF image, from the EM-CCD camera, was loaded with ImageJ. The ECL integral (i.e., pixels intensity) was measured for the bead emission with a square of 30×30 px. The same procedure was used to measure the background emission where beads were not present (average of 4 different points). The ECL integral from beads subtracted by the background is the value used in Fig. 2.

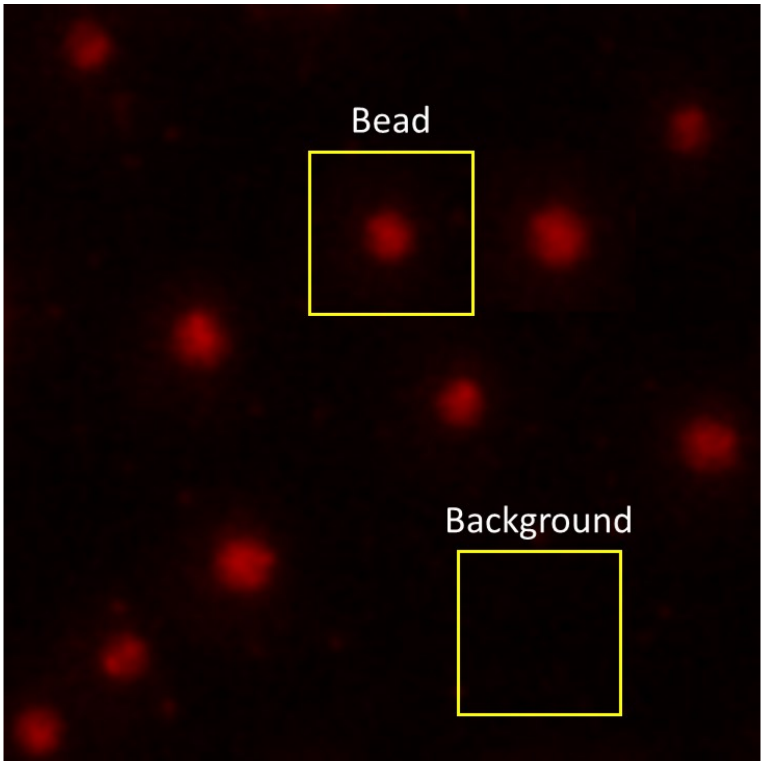

Fig. S3 Example of integration procedure of ECL emission.

Side-view ECL imaging: The electrochemical set-up includes a glassy carbon working electrode, Ag/AgCl (KCl, 3M) reference electrode and Pt wire counter electrode connected to a Metrohm µAutolab type III potentiostat.

A modified epifluorescence microscope (Olympus), as described previously,[3] was used to acquire the PL and ECL images by a 50x microscope objective and an EM-CCD (Hamamatsu).

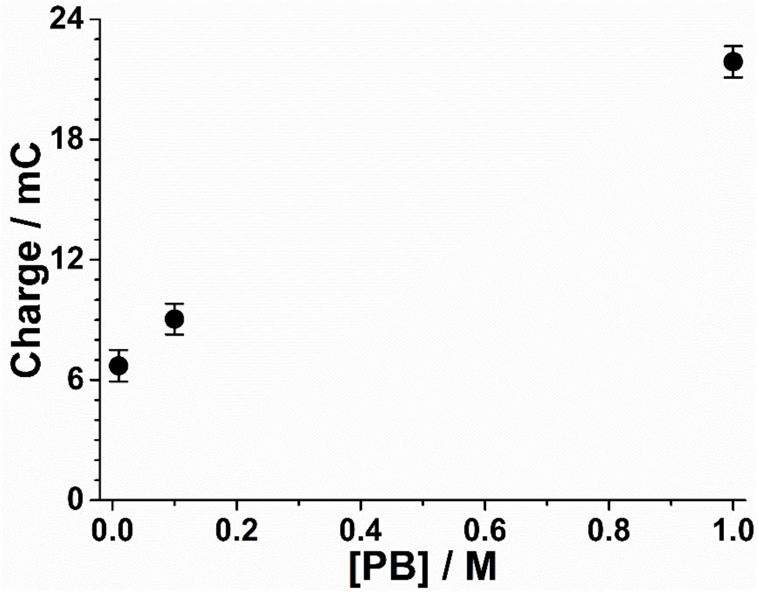

Fig. S4 Charge as function of PB concentration (0.01, 0.1, and 1 M) integrated from chronoamperometry of top-view imaging of beads. Applied potential: 1.4 V vs. Ag/AgCl, time 6 seconds.

References

1 Valenti, G.; Zangheri, M.; Sansaloni, S. E.; Mirasoli, M.; Penicaud, A.; Roda, A.; Paolucci, F. Transparent Carbon Nanotube Network for Efficient Electrochemiluminescence Devices. *Chem. Eur. J.* **2015**, *21*, 12640-12645.

2 Sentic, M.; Milutinovic, M.; Kanoufi, F.; Manojlovic, D.; Arbault, S.; Sojic, N. Mapping electrogenerated chemiluminescence reactivity in space: mechanistic insight into model systems used in immunoassays. *Chem. Sci.* **2014**, *5*, 2568-2572.

3 Chovin, A.; Garrigue, P.; Vinatier, P.; Sojic, N. Development of an Ordered Array of Optoelectrochemical Individually Readable Sensors with Submicrometer Dimensions: Application to Remote Electrochemiluminescence Imaging. *Anal. Chem.* **2004**, *76*, 357-364.
